# Fiber-Degrading Microorganisms: Types, Screening and Applications

**DOI:** 10.3390/life16061014

**Published:** 2026-06-17

**Authors:** Haiying Yang, Baoyan Yang, Wenjie Zhang, Mengrong Su, Qindan Dai, Jian Ma

**Affiliations:** 1 College of Coastal Agricultural Sciences, Guangdong Ocean University, Zhanjiang 524088, China; yanghy738@stu.edu.cn (H.Y.);; 2 Key Laboratory of Livestock and Poultry Healthy Breeding Technology in Northwest China, Xinjiang Agricultural Vocational and Technical University, Changji 831100, China; 3 Sichuan Institute of Musk Deer Breeding, Chengdu 611845, China

**Keywords:** cellulase, lignocellulose degradation, CAZymes, microbial fermentation, feed additives, animal nutrition

## Abstract

Fiber-degrading microorganisms are widely recognized for their potential to convert renewable lignocellulosic biomass into animal feed. However, translating this potential into practical application faces five critical yet underappreciated challenges. First, current screening methods, primarily including plate dilution and Congo red staining, are low-throughput, poorly reproducible and fail to capture the synergistic actions of natural microbial consortia. Second, the lack of standardized assays for quantifying cellulolytic activity compromises the reliability of cross-study comparisons. Third, safety assessments for fiber-degrading microorganisms remain superficial, with most studies neglecting mycotoxin production, antibiotic resistance gene transfer and long-term colonization risks. Fourth, fundamental differences between fungal and bacterial degradative systems, such as enzyme multiplicity, oxygen requirements and cellulosome assembly, are rarely considered in strain selection, leading to suboptimal application outcomes. Finally, the vast majority of positive in vitro degradation results fail to translate into improved animal performance in vivo, owing to poor microbial survival in the gastrointestinal tract, mismatched enzyme activity with gut pH and temperature, coupled with the absence of dose–response validation. This review critically evaluates these five bottlenecks across fiber-degrading microorganism types, screening platforms and practical livestock production applications. Overall, future progress should depend less on discovering “novel” strains and more on establishing standardized screening pipelines, rigorous safety frameworks and mechanistic understanding of in vivo efficacy, including direct head-to-head comparisons between fungal enzymes and bacterial probiotics under identical conditions.

## 1. Introduction

In recent years, against the backdrop of an increasingly tense international situation and the growing competition for grain between humans and livestock, the supply cost of conventional feed resources such as maize and soybean meal has been rising significantly. Consequently, developing new and high-quality feed resources has become an inevitable choice for the sustainable development of the livestock industry [1]. Agriculturally intensive countries are rich in non-conventional feed resources, with vast reserves of food processing by-products (e.g., pineapple pulp), which hold enormous potential for development [2]. However, these resources generally contain high proportions of structural carbohydrates, such as cellulose, resulting in poor palatability and low digestibility, which severely limit their efficient utilization in feed. The efficient conversion of such fibrous resources would not only alleviate the supply pressure of conventional feed ingredients but also significantly enhance the overall utilization efficiency of feed resources, holding important strategic significance for ensuring the stable development of animal husbandry. Currently, the development of fibrous resources primarily relies on microbial degradation and enzymatic hydrolysis technologies.

In animal husbandry, fiber-degrading microorganisms and their enzyme preparations have become key tools for improving the utilization efficiency of fibrous feed. Recently, a study has shown that in a co-fermentation system of corn straw and distillers dried grains with solubles, *Trichoderma reesei* achieves a maximum cellulose degradation rate of 15.7%, while *Aspergillus niger* achieves a hemicellulose degradation rate of 19.4% [3]. In the production of dairy cows, treating forage with cellulase preparations increases feed intake and milk yield, confirming that cellulase, as a feed additive, can improve feed utilization and animal production performance through enhanced fiber degradation [4]. Also, the functional oligosaccharides and organic acids produced during their degrading process help to regulate the intestinal microbiota and enhance the immune function of animals [5]. A previous study conducted a systematic techno-economic analysis of cellulase costs in lignocellulosic biofuel production and identified cellulase production cost as a primary bottleneck limiting large-scale commercialization. According to their economic model, cellulase costs were estimated at $0.68–1.47 per gallon of ethanol produced, equivalent to approximately €5–10 per kilogram of cellulase [6]. Compared with physicochemical pretreatment methods such as acidification and steam explosion, microbial degradation under controlled laboratory conditions avoids the production of inhibitory by-products (e.g., furfural and 5-hydroxymethylfurfural). However, claims of lower energy consumption remain indirect, as most studies have not directly measured energy use under standardized conditions. It also has limitations, including a longer treatment cycle and insufficient enzyme system stability under industrial-scale conditions [7]. This paper presents a systematic review of the main types of fiber-degrading microorganisms, methods for their isolation and screening, and the applications of fiber-degrading microorganisms in livestock and poultry production, aiming to provide theoretical guidance and scientific evidence for advancing the utilization of fiber-degrading microorganisms in the livestock and poultry industry.

## 2. Types of Fiber-Degrading Microorganisms

### 2.1. Fiber-Degrading Fungi

#### 2.1.1. Trichoderma

**Figure 1:**
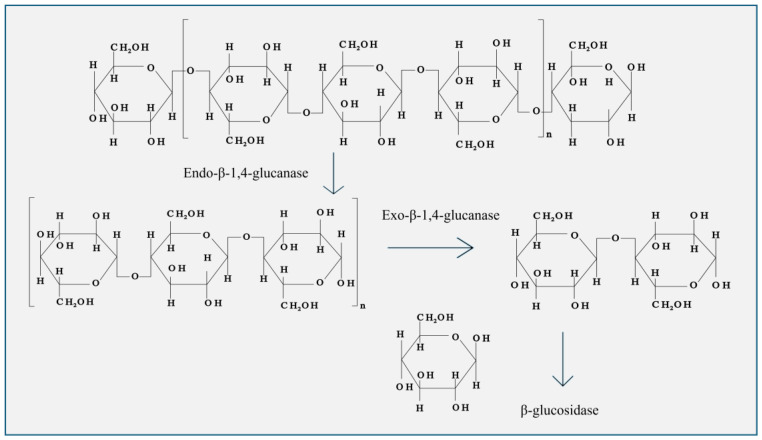
Schematic diagram of the synergistic mechanism of cellulase in cellulose degradation [19].

In natural ecosystems, fungi play a dominant role in the degradation of lignin, hemicellulose and cellulose, and they mediate biochemical degradation through the secretion of extracellular enzymes, while the physical penetration of fungal hyphae synergistically promotes this degradative process [8]. At present, the most widely studied fiber-degrading fungi include the genera *Trichoderma* and *Aspergillus* [9]. As highly ecologically adaptable filamentous fungi, *Trichoderma* species are capable of forming a highly efficient and finely regulated cellulolytic metabolic pathway through the coordinated secretion of a cellulase system comprising endo-β-1,4-glucanases, exo-β-1,4-glucanases and β-glucosidases [10]. As shown in Figure 1, endo-enzymes act on the amorphous regions of cellulose, cleaving internal β-1,4-glycosidic bonds, whereas exo-enzymes target the crystalline regions and release cellobiose from the non-reducing ends. β-glucosidases subsequently hydrolyze cellobiose into glucose, thereby achieving efficient cellulose degradation [11]. These enzymes belong to the broader class of carbohydrate-active enzymes (CAZymes), which are systematically classified into families such as glycoside hydrolases (GH), polysaccharide lyases (PL), carbohydrate esterases (CE) and auxiliary activities (AA) [12]. Among these, lytic polysaccharide monooxygenases (LPMOs, classified under AA families AA9, AA10, AA11) have fundamentally revised the classical model of cellulose degradation. Unlike traditional hydrolases, lytic polysaccharide monooxygenases (LPMOs) are copper-dependent oxidative enzymes that cleave cellulose chains oxidatively, creating new chain ends that serve as priming sites for processive cellulases and increasing overall saccharification efficiency by 2- to 10-fold on crystalline cellulose [13].

Despite the high catalytic efficiency of the *Trichoderma* cellulase system, its application in fiber degradation faces several critical limitations. First, the efficiency of any exogenous enzyme system is constrained by substrate accessibility, namely the physical availability of cellulose surfaces for enzymatic attack. Lignin, as a complex aromatic polymer, acts as a protective barrier that causes cellulase inactivation through non-specific adsorption and blocks access to cellulose fibers, while high cellulose crystallinity further reduces digestibility [14]. This explains why fibrous feed materials generally require pretreatment such as grinding and alkali treatment before or during microbial fermentation to improve substrate accessibility. Second, most current studies remain limited to chemical composition analysis, without assessing the digestibility of fermented feed or animal performance. The genetic stability and safety of mutant strains are unevaluated. Third, in vitro enzyme assays using purified substrates under standard conditions differ greatly from the complex rumen environment, meaning that high in vitro activity may not translate into efficient in vivo degradation. Fourth, subsequent studies should integrate multiple approaches: proteomics to identify the enzymes actually expressed by *Trichoderma* strains on specific substrates (e.g., cellulases and LPMOs); metabolomics to reveal the functional metabolites produced during fiber degradation (e.g., oligosaccharides and organic acids) and standardized in vivo digestion studies to validate whether in vitro results translate into improved performance in practical production, which will provide mechanistic guidance for understanding enzyme synergy and designing efficient enzyme preparations.

Previous research has shown that the rapid production of cellulases by *Trichoderma* fungi facilitates the hydrolysis of lignocellulosic materials, providing a promising resource for ruminant feed supplements [15]. Francesco et al. [16] evaluated the in vitro fiber-degrading capabilities of three *Trichoderma* strains, including *T. atroviride* P1, *T. afroharzianum* T22, and *T. reesei* T67, and results showed that the enzyme mixture produced by strain T22, containing glucanase, cellulase and xylanase, exhibited the highest fiber-degrading activity. When supplemented into ruminant feed, it reduced the content of hemicellulose, cellulose and lignin, and improved fiber degradability and feed efficiency. In poultry, Perim et al. [17] found that the addition of *Trichoderma reesei* cellulase (liquid form, 500 mL/t) to the diet of broiler chickens aged from 1 to 21 days effectively degraded the dietary cellulose, increased fiber digestibility by up to 41.74%, improved nitrogen retention by 44.6% and reduced nitrogen excretion. In the context of solid-state fermentation, Saa et al. [18] evaluated the potential of the mutant strain *Trichoderma viride* M5-2 to improve the nutritional composition of lentils and hairy vetch through solid-state fermentation and found that this mutant strain increased the crude protein content of both plants while reducing fiber content.

#### 2.1.2. Aspergillus

In addition to *Trichoderma*, fungi of the genus *Aspergillus* (e.g., *Aspergillus niger*) are also representative strains capable of highly efficient enzyme production. The cellulase produced by *Aspergillus* is a multi-enzyme complex comprising endo-β-glucanase, exo-β-glucanase and β-glucosidase, which can efficiently break down cellulose into small-molecule substances that can be utilized by livestock and poultry [20]. Cellulose is a linear polymer composed of numerous D-glucose units linked by β-1,4-glycosidic bonds, which serves as the specific target of these enzymes, thereby enabling efficient cellulose degradation by the enzymatic system [21]. Abdusamatov et al. [22] screened fungal strains for multi-enzyme production and reported that *Aspergillus* strain N-17 exhibited rapid growth and robust enzyme-producing capacity. This strain could efficiently and simultaneously secrete proteases, xylanases, and cellulases, with its endo-1,4-β-glucanase (3.8–4.1 U/mL), exo-1,4-β-glucanase (2.9–3.1 U/mL), and xylanase (7.2–7.5 U/mL) activities being significantly higher than those of other tested strains, enabling the effective conversion of cellulose into products rich in carbohydrates and proteins. As an important industrial filamentous fungus, *Aspergillus niger* is not only capable of effectively degrading plant fiber components but also secretes various organic acids and enzyme preparations, thereby enhancing the nutritional value of feed. Khasanah et al. [23] evaluated the solid-state fermentation of coffee husks using fungi including *Aspergillus niger*, *Trichoderma harzianum* and *Saccharomyces cerevisiae*, and results showed that this fermentation significantly reduced crude fiber and lignin content while increasing total digestible nutrients, thereby improving the nutritional value of coffee husks as ruminant feed. Furthermore, Afeni et al. [24] investigated the effect of *Aspergillus niger* fermentation on the bioavailability of copper and zinc in three fibrous feeds (wheat bran, brewer’s grains and palm kernel meal), as well as its regulatory role on the production performance and serum cholesterol levels of growing pigs, and results showed that fermentation significantly improved the bioavailability of copper and zinc in the feed (with increases of 53.35%, 7.90% and 4.54%, respectively) and effectively reduced total serum cholesterol levels in growing pigs by approximately 30%. Among the treatments, fermented brewer’s grains demonstrated the best growth-promoting effect, whereas fermented palm kernel meal exhibited the most favorable cholesterol-lowering effect.

However, the application of *Aspergillus* in feed necessitates rigorous biosafety considerations. Firstly, some strains produce mycotoxins (e.g., aflatoxins), requiring strain-level identification and verification as non-toxigenic. Secondly, regulatory safety designations (e.g., GRAS in UAS and QPS in EU) are strictly strain-specific rather than applicable to entire species. Thirdly, robust quality control protocols are required to monitor mycotoxin contamination, genetic stability of production strains, and batch-to-batch consistency of fermentation products. Notably, these issues are rarely reported in current studies. Future study should prioritize comprehensive safety evaluations in parallel with industrial performance validation. From a mechanistic perspective, the cellulase system of *Aspergillus niger* involves multiple CAZyme families, and its gene expression is tightly controlled by a regulatory network composed of transcriptional activators and repressors [25]. Unfortunately, the actual role of this network in the animal gut remains largely unexplored.

In summary, existing evidence, derived largely from laboratory-scale studies, suggests that *Trichoderma* and *Aspergillus* represent promising candidates for improving fiber utilization in the feed industry, offering two complementary approaches. As exogenous enzyme additives, enzyme systems from *Trichoderma* have demonstrated effective fiber degradation potential in vitro, though their precise in vivo mode of action remains to be fully elucidated. *Aspergillus* has shown utility primarily in the bioconversion of feed materials. However, it must be emphasized that the current understanding is predominantly based on small-scale studies. Robust industrial validation and comprehensive economic feasibility assessments are critically lacking. The combined application of these microorganisms represents a valuable research direction, but its practical potential remains contingent upon further large-scale validation and economic evaluation.

### 2.2. Fiber-Degrading Bacteria

#### 2.2.1. Bacillus

Compared with fungi, fiber-degrading bacteria generally produce a more limited spectrum of cellulolytic enzymes and may present certain limitations for the large-scale production of highly active enzyme preparations. However, their most prominent strength lies in their widespread ecological distribution, rendering them a valuable resource for the discovery of cellulases with specialized functions [26]. The most frequently reported fiber-degrading bacteria currently include the genera *Bacillus* and *Streptomyces* [27,28]. Among these, *Bacillus* can sense environmental signals reflecting carbon source limitation or the presence of cellulose-like substrates. Through intracellular regulatory networks, they activate cellulase-encoding genes, synthesize cellulase proteins via transcription and translation, and secrete these enzymes extracellularly through dedicated secretion systems to degrade cellulose [29]. The main stages and key characteristics of cellulose degradation by *Bacillus* species are shown in Table 1. A previous study by Akhtar et al. [30] confirmed that *Bacillus subtilis* CP-9, isolated from camel feces, secretes cellulase, xylanase and amylase. Following 48 h of fermentation with soybean meal, this strain degraded antinutritional factors, reduced neutral detergent fiber content by 34.25% and increased crude protein content by 16.54%, thereby significantly improving the ileal digestibility of dry matter, crude protein and fiber in growing pigs.

The conversion of food industry by-products (e.g., citric acid residue) into animal feed is constrained by nutritional limitations, among which high fiber content represents the primary limiting factor. To this end, Tanpong et al. [31] evaluated changes in the nutritional value of citric acid by-products fermented using *Bacillus subtilis* I9, and results showed that fiber content in the fermented product was decreased, crude protein was increased by 21.89%, and the amino acid profile was notably improved, which indicated that fermentation of citric acid by-products by this strain can effectively reduce dietary fiber content and improve the nutritional characteristics of the by-products, making them suitable for animal feed. Khongkool et al. [32] isolated spore-forming *Bacillus amyloliquefaciens* strains from fecal samples of free-range indigenous pigs raised on farms and after screening and characterizing the isolates, the most promising strain, *Bacillus amyloliquefaciens* NL1.2, was evaluated for in vivo safety and efficacy in a mouse model. The results revealed that this bacterium can produce firolytic enzymes and significantly increased intestinal secretory immunoglobulin A levels and the abundance of probiotics (e.g., *Bacteroides phylum*) while reducing the abundance of pathogenic bacteria (e.g., *Helicobacter* spp.). Currently, research on *Bacillus* species as cellulose-degrading bacteria has largely focused on *Bacillus subtilis*, whereas the cellulolytic potential of other congeneric species remains relatively underexplored. Notably, despite *Bacillus* species producing free extracellular cellulases, many anaerobic bacteria (e.g., *Clostridium* and *Ruminococcus*) employ a distinct supramolecular machinery called the cellulosome, and it is a multi-enzyme complex assembled via cohesin–dockerin interactions that promotes substrate channeling and inter-enzyme synergy [33]. Although cellulosomes are less common in *Bacillus*, the principle of enzyme synergy is increasingly recognized as a key determinant of degradation efficiency in complex environments such as the animal gut.

#### 2.2.2. Streptomyces

*Streptomyces* actinobacteria constitute a group of microorganisms capable of degrading a variety of recalcitrant substrates and play a significant role in the global carbon cycle. Priyadarshani et al. [34] isolated two actinobacterial strains, *Streptomyces thermoviolaceus* S1 and S2, from compost via enrichment culture, and both strains demonstrated the ability to produce lignocellulolytic enzymes and could degrade the lignocellulosic matrix when cultivated on rice straw under solid-state fermentation. Compared with S1, strain S2 exhibited superior lignin removal efficiency and higher cellulase activity. Melo et al. [35] screened two *Streptomyces* strains (F1 and F7) from soil that exhibited high endoglucanase and endochitinase activities. When cultivated on steam-pretreated bagasse as the substrate, both strains displayed a broad spectrum of CAZyme activities and were capable of degrading various substrates, including arabinans, xylans, β-glucans, starch, CMC and chitin. Among them, strain F7 could additionally degrade pectin, mannan and lichenans. Furthermore, Ozkan [36] demonstrated optimal synergistic effects through the co-cultivation of *Streptomyces* strains OZ2 and OZ5, with cellulase activity reaching 26 U/mL, which exceeded that of any monoculture. By optimizing the fermentation conditions (3 g/L yeast extract, 60 h, pH 6.0 and 35 °C), cellulase activity was enhanced to 56 U/mL, representing an approximately 3.5-fold increase relative to the initial level.

Overall, fungi, including *Trichoderma* and *Aspergillus*, are primarily used in the feed industry as high-efficiency producers of extracellular enzymes, directly breaking down fiber and releasing nutrients in the form of added enzyme preparations. Bacteria, on the other hand, are more commonly used as live fermentation inoculants or probiotics, which pre-treat feed through metabolic activities or directly modulate the host’s gut microbiota. Although the two differ fundamentally in their mechanisms of action, application approaches and dosages, they show strong mutual complementarity. Although *Bacillus* and *Streptomyces* possess inherent advantages in spore formation and ecological adaptability, they still face three prominent challenges: the respective contributions to fiber degradation and immunomodulation remain poorly defined; a lack of unified methodological standards hinders reliable cross-study comparisons; and their intrinsic enzyme activity is relatively low and seldom systematically optimized. Furthermore, direct head-to-head comparisons between fungal enzymes and bacterial probiotics are lacking. Although both approaches have been studied independently, few studies have systematically compared their efficacy under identical experimental conditions using consistent animal models, diets, dosage rationale and outcome measures. This gap is particularly critical, and without such comparisons, it remains unclear whether fungal enzymes, bacterial probiotics or their combination is more effective or cost-efficient for specific applications (e.g., different animal species or dietary fiber levels). Therefore, conducting controlled comparative trials to fill this gap should be a current research priority.

Taken together, different fiber-degrading microorganisms exhibit marked differences in strain characteristics, enzyme systems, environmental adaptability and industrial applications. In addition, the choice of screening methods and host species directly influences fiber degradation efficacy. To facilitate systematic comparison, Table 2 and Table 3 summarize these aspects from the perspectives of microbial taxa, screening methods and host species.

## 3. Screening of Fiber-Degrading Microorganisms

### 3.1. Plate Dilution Method

The plate dilution method is a reliable basic technique for microbial quantification, and it involves serially diluting samples and culturing them on agar plates to form distinct and countable single colonies, thereby enabling microbial isolation, enumeration of viable cells as Colony-Forming Units (CFUs), and assessment of microbial viability. This method is simple and low-cost, but it has low sensitivity and moderate reproducibility that is highly dependent on experimental conditions and low throughput. In major grain-producing nations, straw resources are abundantly available, yet conventional treatment methods are inefficient and carry environmental risks, and the key to achieving efficient degradation lies in the screening of specialized microorganisms. Recently, there has been growing interest in the exploration of cold-adapted microorganisms, as they can address the challenge of corn straw degradation under low-temperature conditions, thereby providing new pathways for sustainable agricultural development. He et al. [37] used the plate streaking method to isolate a highly efficient, cold-tolerant cellulolytic bacterium at 10 °C, which was identified as *Bacillus subtilis* K1. Genomic sequencing and annotation of this strain revealed a total of 4213 genes, including numerous functional genes associated with lignocellulose degradation. Enzymatic activity assays showed that the strain exhibited strong cellulolytic activity, with maximum endo-β-glucanase, exo-β-glucanase and β-glucosidase activities reaching 24.69 U/mL, 1.72 U/mL and 1.14 U/mL, respectively. When strain K1 was applied to maize straw composting at 6 °C, the average compost temperature increased by 86.7% compared with the control, and the ratio of humic acid to fulvic acid (HA/FA) increased by 94.02%, while the lignocellulose degradation rate decreased by 18.01–41.39%. This study provides a solid theoretical foundation for the application of cold-tolerant cellulolytic bacteria in the resource utilization of corn straw. Furthermore, Muhammadn et al. [38] isolated a strain of *Cellulomonas* sp. ASN2 from soil using the serial dilution and pour plate methods, and the crude enzyme produced by this strain exhibited optimal activity at pH 7.5 and 60 °C. Co^2+^ and Mn^2+^ were found to exert activating effects on the enzyme, suggesting its promising potential for use in the saccharification of plant biomass for bioethanol production. The plate dilution method serves as a critical preliminary screening approach for isolating fiber-degrading microorganisms, yet it presents clear limitations: it relies on standardized laboratory conditions, making it difficult to faithfully reproduce the heterogeneity of natural habitats; furthermore, it focuses on pure-strain isolation and thus overlooks the synergistic degradation effects within microbial communities. To address these two major limitations, several technical improvements have been proposed, such as enrichment culture methods, in situ culture techniques, co-culture screening strategies and metagenomics-guided targeted isolation. Nevertheless, these improvements still rely predominantly on the plate-based isolation procedures and cannot fully represent the full spectrum of microbial diversity in natural ecosystems. The plate dilution method is primarily applicable to enzyme activity screening and initial isolation of candidates for live microbial inoculants, but it does not provide information on probiotic traits (e.g., immunomodulation and pathogen inhibition) or industrial fermentation stability (e.g., batch-to-batch consistency and downstream processing stability).

### 3.2. Congo Red Staining Method

The Congo red staining method is a technique that relies on the specific binding of Congo red to cellulose to form a red complex, and its function is to identify the activity of fiber-degrading microorganisms by observing the presence or absence of clear hydrolysis zones. This method has moderately low reproducibility and semi-quantitative sensitivity, cannot provide precise quantification, and is only suitable for preliminary screening. In addition, it cannot distinguish the cellulase types and may produce false results but remains widely used for rapid visual screening. Chen et al. [39] employed the Congo red staining method to screen and optimize the high-enzyme-producing purple *bacillus* CMK-7, raising its enzyme activity from 289.12 to 332.95 U/mL. This strain was then combined with the nitrogen-fixing bacterium LMY-3-2 at a 1:1 ratio to form the microbial inoculant A7. In buckwheat experiments, the plant height growth of the A7-treated group increased by 96.5% and 193.9% compared to the sterile water control and the single nitrogen-fixing bacterium treatment, respectively. This system simultaneously achieved waste degradation, soil amelioration and crop yield enhancement, providing an efficient microbial strategy for advancing the agricultural circular economy. Mokale et al. [40] isolated six highly efficient fiber-degrading microorganism strains from soil using the Congo red staining method. Using the dinitrosalicylic acid assay and response surface methodology, together with optimization of various environmental and nutritional parameters, they significantly enhanced the cellulase activity of these strains, achieving a maximum activity of 190.30 U/mL. These strains represent promising biocatalysts for the industrial conversion of cellulose into glucose. Furthermore, Li et al. [41] screened and identified a strain of acid- and heat-tolerant *Bacillus subtilis* DC-11 from silkworm feces. After preliminary screening via CMC-Na medium culture combined with Congo red staining, the strain was rescreened using filter paper activity assays, accompanied by morphological observation, physiological and biochemical characterization, and phylogenetic analysis. This strain exhibited optimal cellulase activity at pH 6.0 and 55 °C, with a filter paper activity of 15.40 U/mL. When applied, it significantly elevated the cellulose degradation rate from 10.01 to 39.57%, making it an excellent candidate for rapid composting of agricultural wastes such as silkworm manure. Both the Congo red staining and plate dilution methods are suitable only for enzyme activity screening and cannot assess a strain’s survival capacity as a live microbial inoculant, probiotic traits and industrial production stability.

### 3.3. Genomics

In research on fiber-degrading microorganisms, genomics analysis is highly sensitive and efficient, capable of retrieving genetic information from unculturable microorganisms, but gene presence does not guarantee actual enzymatic activity, which depends on bioinformatics expertise. Thus, it is suitable for functional gene mining rather than activity validation. Genomics and related omics technologies have evolved from single whole-genome sequencing into a multi-layered integrated analytical framework encompassing comparative genomics, metagenomics, metatranscriptomics and metagenome-assembled genome (MAG) reconstruction [42]. Comparative genomics systematically identifies CAZyme families, such as glycoside hydrolases (GH) and polysaccharide lyases (PL), through CAZyme annotation pipelines, thereby revealing differences in fiber-degrading gene clusters among strains and their ecological adaptations. However, several technical limitations must be considered when using these annotation tools. First, the CAZyme annotation is heavily dependent on the quality and completeness of the reference database, and novel or highly divergent CAZyme families may be missed. Second, these pipelines predict genetic potential based on sequence homology, but they cannot confirm whether a predicted CAZyme gene is actually expressed or catalytically active under physiological conditions. Beyond the limitations of CAZyme annotation, methods such as metagenomics themselves face technical challenges. Metagenomics enables direct DNA extraction and sequencing from environmental samples without cultivation and, combined with binning techniques, facilitates MAG reconstruction to obtain genomic information from unculturable fiber-degrading microorganisms, with quality ensured by completeness assessment tools such as *CheckM* [43]. Although MAG reconstruction has revolutionized the study of unculturable microbes, it faces three major challenges. Firstly, binning accuracy: different binning algorithms (e.g., MetaBAT, MaxBin and CONCOCT) can yield different genome reconstructions from the same dataset, and the choice of algorithm significantly affects downstream conclusions. Secondly, contamination: MAGs frequently contain sequences from closely related species or even unrelated taxa, and high contamination rates can severely compromise the reliability of functional predictions. Lastly, completeness: low-completeness MAGs may lack essential genes required for fiber degradation, leading to false-negative conclusions. Tools such as CheckM and BUSCO are commonly used to assess MAG quality, but current standards for acceptable completeness and contamination thresholds vary across studies, limiting cross-comparison.

Metatranscriptomics, by sequencing total RNA from environmental samples, identifies CAZyme genes that are actually expressed during fiber degradation, thereby dynamically linking genetic potential to actual functionality. Metatranscriptomics faces its own challenges, including RNA instability during sample handling, the need for efficient rRNA depletion and difficulties in assigning transcripts to specific microbial species in complex communities. Nevertheless, these methods share common limitations: genomics and metagenomics predict genetic potential rather than actual enzymatic activity; metatranscriptomics faces technical challenges such as RNA instability and high-abundance rRNA interference and MAG completeness and contamination rates directly affect the reliability of subsequent conclusions. A critical step that remains underutilized is functional validation. Ideally, CAZyme genes predicted from genomic or metagenomic data should be cloned, heterologously expressed and biochemically characterized to confirm enzyme activity, substrate specificity and kinetic parameters. Without such validation, in silico predictions remain hypotheses rather than confirmed findings. Integrated multi-omics strategies, for example, identifying CAZyme gene pools via metagenomics, confirming actively expressed genes via metatranscriptomics, and then dissecting the genetic features of key strains through comparative genomics have become a frontier direction in current research.

Importantly, omics tools can serve all four screening categories: enzyme activity screening benefits from metaproteomics; live inoculant screening benefits from metatranscriptomics; probiotic trait screening benefits from metabolomics; and industrial stability screening benefits from comparative genomics. Zhang et al. [44] performed whole-genome sequencing and analysis on the cellulolytic strain *Bacillus subtilis* DC11 isolated from silkworm feces. They identified the key cellulase gene *ytoP*, which was subsequently cloned and heterologously expressed in *Escherichia coli*. This gene encodes a protein of approximately 40–50 kDa, and after purification, its enzymatic activity reached 12.98 U/mL, laying a solid foundation for the large-scale application of microbial cellulose degradation. Furthermore, Zhang et al. [45] used genome sequencing to characterize *Bacillus velezensis* SSF6, which possesses a 3.89 Mb genome. The strain was found to harbor an abundance of genes related to carbohydrate metabolism and hydrolase synthesis. Enzymatic assays further confirmed its high cellulolytic activity, with filter paper activity of 64.48 U/mL and exo-β-glucanase activity of 78.59 U/mL. These results provide a solid theoretical basis for the application of this strain in cellulose degradation and bioconversion. From a genomic perspective, systematic CAZyme annotation using pipelines such as dbCAN allows researchers to rapidly identify the full repertoire of CAZyme encoded in a bacterial genome. This approach reveals not only cellulases but also hemicellulases, pectinases and auxiliary enzymes that collectively determine a strain’s fiber-degrading potential. However, a critical gap remains: genomic prediction of CAZyme genes does not guarantee their actual expression or activity under gut conditions, necessitating integration with transcriptomic and proteomic validation. Furthermore, functional metagenomics, which involves cloning environmental DNA into expression hosts to directly screen for desired enzymatic activities, offers a powerful complement to sequence-based prediction. Coupled with computational enzyme prediction tools, such as machine learning algorithms trained on CAZyme sequences, these approaches can accelerate the discovery of novel fibrolytic enzymes from uncultured microorganisms without prior sequence homology. A previous study isolated *Bacillus amyloliquefaciens* strain TL106 from Tibetan pig feces, and genomic analysis revealed that this strain harbors multiple genes encoding cellulases and hemicellulases. Its fermentation supernatant effectively degrades arabinan and β-glucan in wheat and highland barley, highlighting its potential as a feed additive to enhance fiber digestibility in monogastric animals [46].

### 3.4. Isolated Cultures and Omics

The integration of traditional isolation and screening methods with the multi-omics technologies described above provides complementary advantages and achieves combined functional and mechanistic analysis. Metagenomics predicts genetic potential, metatranscriptomics captures gene expression, metaproteomics provides direct evidence of enzyme abundance and metabolomics reflects the functional outcomes of degradation [47,48]. Currently, function-driven isolation strategies are a major research focus. A standardized workflow can be established as follows: in the early phase, culturable strains are isolated using plate-based methods while establishing a metagenomic baseline; in the intermediate phase, active functional taxa are identified using metatranscriptomics, metaproteomics, or both; in the late phase, targeted isolation of candidate strains is performed, followed by whole-genome sequencing, comparative genomics and biochemical validation [49]. However, it is important to distinguish between what has been actually achieved and what remains a future prospect. To date, most studies in the feed sector have successfully applied plate-based isolation combined with 16S rRNA sequencing or whole-genome sequencing of individual isolates. In contrast, the routine integration of metatranscriptomics, metaproteomics and metabolomics into fiber-degrading microorganism research for feed applications remains largely at the proof-of-concept or promising stage. Alternative pathways may also be adopted depending on research objectives. Wang et al. [50] employed the dilution plating method coupled with Congo red staining to isolate a novel *Pseudoxanthomonas* strain JC1303 from marine sediments. Using whole-genome sequencing and transcriptomic analysis, they elucidated its complete cellulolytic system and metabolic versatility. This strain exhibits distinctive genomic features, including an open pan-genome and a large number of strain-specific genes. Under cellulose induction, it realizes efficient coupling between extracellular hydrolysis and substrate uptake by upregulating key endoglucanase and membrane transporter genes. This study provides a solid theoretical foundation for deciphering the cellulose degradation mechanisms of marine bacteria and for developing high-efficiency biocatalysts for cellulose bioconversion.

In addition, a previous study isolated and screened *Bacillus subtilis* K35-1 from yak rumen fluid using Congo red staining. Its maximum cellulase activity reached 77.26 U/mL, and the feed cellulose degradation rate was as high as 53.2%, compared with only 7.3% in the conventional treatment group. Comparative genomic analysis revealed that the glycosidase gene abundance in this strain was 40% higher than in other reference strains, and it exhibited almost no antibiotic resistance [51]. As a highly efficient and safe degrading candidate, this strain provides a valuable genetic basis for the development of novel feed additives. In China, the annual output of distiller’s grains is substantial. However, their high fiber and low nutrient content pose challenges for efficient utilization [52]. Microbial fermentation is a key strategy to enhance their feeding value. Nevertheless, suitable microbial strains remain scarce, highlighting the urgent need to screen and develop novel and efficient fermentation strains for this industry. In preliminary work, our team isolated and characterized a fiber-degrading bacterial strain, *Cohnella xylanilytica* T5, from humus soil using the plate method, followed by 16S rRNA sequencing and morphological analysis. Fermentation of distiller’s grains with this strain effectively disrupted the grain structure, degraded fiber and increased protein content, indicating its potential as a candidate strain for developing distiller’s grain-based feed [53]. However, the activity of such efficient in vitro degraders may be suppressed within the complex digestive tract environment of livestock and poultry (e.g., due to pH variations), necessitating further investigation. Beyond omics-based discovery, advanced genetic engineering tools such as CRISPR-Cas systems have enabled precise genome editing of fiber-degrading microorganisms, allowing targeted knockout of repressive regulators, overexpression of rate-limiting enzymes, or integration of heterologous CAZyme genes into robust chassis strains. These genetic engineering approaches have rarely been applied to fiber-degrading microorganisms intended for animal feed applications, and most reports remain at the laboratory scale using model organisms such as *Escherichia coli* or *Saccharomyces cerevisiae.* Furthermore, artificial intelligence (AI)-assisted enzyme discovery is emerging as a transformative approach, where deep learning models (e.g., protein language models) predict enzyme function, thermostability and substrate specificity from sequence data alone, dramatically reducing the need for labor-intensive experimental screening. Integrating AI predictions with experimental validation represents a promising frontier for rational design of fibrolytic enzymes and strains.

In conclusion, traditional plate dilution and Congo red staining methods are simple and inexpensive but are suitable only for enzyme activity screening. They cannot meet the requirements for screening live microbial inoculants, probiotic traits, or industrial stability, nor can they capture microbial community dynamics. Multi-omics technologies provide complementary information across all four screening categories, but each method has its own limitations, and several specific limitations of omics-based approaches warrant consideration. First, CAZyme annotation pipelines are prone to errors, including false-positive predictions due to sequence homology mismatches, as well as false-negative results when genes belong to novel or highly divergent families that are not represented in reference databases. Second, existing CAZyme databases (e.g., dbCAN and CAZy) are inherently incomplete, particularly for understudied microbial taxa or environments, which can lead to a systematic underestimation of a strain’s fiber-degrading potential. Third, and most critically, in silico predictions based on genomic or metagenomic data cannot confirm actual enzymatic activity, substrate specificity or expression under gut-relevant conditions. Therefore, functional experimental validations, including heterologous expression, biochemical characterization and activity assays, remain essential for confirming predictions and should not be replaced by purely computational approaches. Critically, while plate-based isolation combined with whole-genome sequencing of individual isolates has been widely and successfully applied in feed-related research, the integration of metatranscriptomics, metaproteomics, metabolomics, CRISPR engineering and AI-assisted discovery into routine workflows for fiber-degrading microorganisms in the feed sector remains largely at the proof-of-concept or exploratory stage, with limited published validation in practical animal production settings. Given that current research remains heavily focused on enzyme activity screening with insufficient attention to the other three categories, future screening should adopt a multi-objective framework tailored to the intended application.

## 4. The Application of Fiber-Degrading Microorganisms in Livestock and Poultry Production

### 4.1. The Application of Fiber-Degrading Microorganisms in Pig Production

Monogastric mammals such as pigs lack endogenous cellulase, rendering them unable to efficiently utilize fibrous resources such as crop straw, which consequently leads to an excessive reliance on conventional feed in intensive livestock production. Microbial fermentation employing fiber-degrading microorganisms enables the conversion of lignocellulose into readily absorbable nutrients. This opens up new avenues for developing cost-effective feed, alleviates human–livestock competition for feed grain, and simultaneously improves intestinal health to further enhance livestock production efficiency. Collectively, existing studies reveal pronounced species-specific differences in strain selection and application outcomes. Wang et al. [54] investigated the effects of feed fermented using a co-culture of *Bacillus subtilis* and *Enterococcus faecium* (FF) on the reproductive performance, immune function and gut microbiota of lactating sows. Compared with the probiotic mixture (PRO) and the basal diet, FF significantly increased feed intake, milk quality and piglet weight gain in sows, while reducing backfat loss, constipation and the incidence of piglet diarrhea. Mechanistically, the beneficial effects of FF are mediated by optimizing gut microbiota composition (enriching *Lactobacillus* and inhibiting *Enterobacteriaceae*), enhancing immune status (elevating IgG and IgM concentrations), promoting anti-inflammatory responses (increasing IL-10 levels) and regulating microbial metabolism. Importantly, FF outperformed PRO, suggesting that its efficacy derives not only from the probiotic strains themselves but also from their metabolites and the fermentation process.

In piglets, a previous study demonstrated that dietary supplementation with *Bacillus subtilis* C-3102 spores significantly increased body weight, average daily gain and feed conversion ratio, and it also improved the digestibility of dry matter, crude protein and gross energy while reducing diarrhea scores in the early post-weaning period, thereby effectively enhancing growth performance in piglets [55]. This study has high reproducibility and well-defined parameters, but its limitations include a single dosage level of spore supplementation and the absence of a control group for fermentation metabolites. Notably, although both studies employed *Bacillus subtilis*-based preparations, their phenotypic outcomes in pig models differed in research emphasis. The former mainly focused on improving growth performance, while the latter placed more emphasis on immunomodulatory effects. These discrepancies may stem from differences in strain specificity, dosage and feeding regimen, but direct comparative studies to verify this remain lacking. In contrast to the direct supplementation strategies described above, Fan et al. [56] optimized the co-fermentation parameters of *Aspergillus niger* and yeast, including fermentation time, inoculation level, solid–liquid ratio and strain ratio, thereby markedly improving the nutritional value of maize by-products. Specifically, crude protein content increased by 14.6% (reaching 32.3 g/100 g), the relative proportion of key amino acids (arginine, threonine and lysine) rose to 13.6%, and cellulose content decreased by 16.5%. In vitro dry matter and protein digestibility also increased dramatically from 35.2% and 32.8% to 53.7% and 74.4%, respectively. This strategy provides technical support for the high-value utilization of maize by-products and the sustainable development of swine feed. Compared with the in vivo studies above, the improvement in digestibility reported in this study was validated only through in vitro assays, without confirmation in animal feeding trials. Furthermore, only one inoculation ratio was tested, and safety assessment was lacking, which highlights the persistent gap between laboratory findings and practical application. Furthermore, Lamu et al. [57] demonstrated that the efficient utilization of high-fiber diets in Tibetan pigs relies on specialized microbial mechanisms in the large intestine, with *Fibrobacter* and the p-75-a5 community serving as key functional taxa. By elevating cellulase and hemicellulase activities, these microbes promote fiber degradation and enhance short-chain fatty acid (SCFA) production, thereby enabling efficient fiber utilization while maintaining normal growth performance and nutrient digestibility. Unlike the exogenous supplementation studies described above, this is an observational study that did not involve any exogenous strain addition or dosage design; therefore, it does not provide information on the application efficacy of exogenous fiber-degrading microorganisms, and its reproducibility cannot be assessed. Nevertheless, this study offers valuable insights into host endogenous mechanisms. Although fiber-degrading microorganisms hold considerable potential for improving feed efficiency and intestinal health, their practical efficacy is modulated by multiple factors, including dietary composition, pig breed, intestinal microbiota structure and growth stage. Elucidating differences in the interactions between gut microbiota and the host across diverse pig breeds, and leveraging these insights to develop more targeted probiotic formulations for optimized application outcomes, are important research directions.

Synthesizing the above studies, pig production studies show marked variation across seven key dimensions: animal model, dosage, substrate, fermentation type, duration and endpoint measures. Relevant studies have validated the efficacy of fiber utilization strategies from four perspectives, namely fermented feed (including metabolites), direct spore supplementation, in vitro process optimization and host endogenous microbiota. However, common limitations include insufficient reproducibility, lack of dosage-response relationships and lack of in vivo validation. Furthermore, direct comparisons across studies are difficult due to differences in strains, dosages and experimental designs, and a persistent gap remains between in vitro optimization and in vivo validation.

### 4.2. The Application of Fiber-Degrading Microorganisms in Poultry Production

The use of fiber-degrading microorganisms to ferment feed has become a key strategy for reducing poultry feed costs and improving resource utilization efficiency. Different studies have shown diversity in microbial strain sources, fermentation substrates and application effects. Feng et al. [58] demonstrated that replacing 5% of dietary corn with wheat bran fermented by *Bacillus cereus* effectively elevated duodenal amylase activity and significantly increased cecal microbial community richness, highlighting its potential to improve intestinal digestive function and maintain microecological homeostasis. However, this study only evaluated a single substitution level (5%) and failed to report key fermentation parameters (temperature, pH and duration) and wheat bran batch-to-batch variability, which limited the independent replication. In addition, it provided insufficient mechanistic insight, as it relied exclusively on 16S rRNA gene sequencing for microbiota profiling without metabolomic or transcriptomic analysis. Hatta et al. [59] demonstrated that dietary supplementation with crude cellulase derived from *Trichoderma viride*-fermented dried coconut pulp significantly improved protein and crude fiber digestibility as well as apparent metabolizable energy in broilers, while lowering cholesterol content in breast muscle. These effects exhibited a curvilinear response with increasing enzyme inclusion levels. Unlike the above study using bacterial fermentation of wheat bran, the present study employed a fungal-derived crude cellulase to treat dried coconut pulp, highlighting the diversity of substrate and microbial sources. Digestive organ weights and breast muscle protein proportion were not significantly altered. Alyileili et al. [60] evaluated the efficacy of degraded date pit (DDP), produced via solid-state fermentation with *Trichoderma reesei*, as an alternative to antibiotics in broiler diets. Dietary supplementation with 10% DDP significantly elevated pancreatic amylase activity, increased intestinal villus height, and reduced crypt depth. Compared with oxytetracycline and mannan oligosaccharides, DDP represents a viable alternative to antibiotic growth promoters in broiler production, effectively improving intestinal development and health. This study has relatively high reproducibility, but the crude protein, fiber and active substances after fermentation were not quantified. Notably, among the studies reviewed, this is one of the few that explicitly compared the test product with antibiotics and commercial prebiotics, providing a more rigorous benchmark.

In the production of laying hens, a previous study demonstrated that dietary supplementation with wheat bran fermented by *Aspergillus niger* and *Aspergillus oryzae* to a low-protein diet (with crude protein reduced by 4.61%) delivers multiple beneficial effects through its antioxidant properties and the complementary action of the two strains, which include decreased phytic acid content, reduced yolk oxidation, and increased egg weight, thereby effectively improving egg quality [61]. However, the above study tested only a single dosage and did not report fermentation parameters or preparation stability, which limits the reproducibility of the results. Lin et al. [62] investigated the effects of *Laetiporus sulphureus* fermentation product (FL) as a feed additive on antioxidant capacity, tight junction gene expression and intestinal morphology in broilers. The results showed that dietary supplementation with FL (especially at 5%) significantly improved feed conversion ratio in broilers. FL optimized intestinal morphology and function by enhancing antioxidant enzyme activities in serum and intestinal tissues, upregulating the expression of tight junction–related genes, including claudin-1 and mucin-2, as well as increasing villus height. These effects collectively improved the antioxidant status and intestinal health of broilers. Notably, this study tested three dosages (2.5%, 5% and 10%) and found that only 5% was effective, revealing the risk that single-dosage testing may miss an effective window. Compared with studies that tested only a single level, this multi-dosage design represents a methodological advantage. However, fermentation conditions and stability data were still not reported. In addition, Wang et al. [63] demonstrated that dietary supplementation with *Bacillus subtilis* DSM 29784 effectively alleviates subclinical necrotic enteritis in broilers. Through synergistic effects across multiple pathways, including improving intestinal architecture, regulating gut microbiota and suppressing cellular apoptosis, this strain enhances growth performance, reduces intestinal lesions, elevates the villus height-to-crypt depth ratio, and increases duodenal maltase activity. It also enriches beneficial microbiota, downregulates the key apoptotic protein caspase-3 in the jejunal mucosa, and lowers the expression of inflammation-associated proteins. Of note, although both *Bacillus cereus* and *Bacillus subtilis* belong to the same genus and have shown intestinal health benefits in poultry models, direct comparability of their results is limited due to the substantial differences in experimental design, outcome measures and dosage levels across the two studies. However, owing to the short length of the avian digestive tract and the rapid transit of digesta, exogenous fiber-degrading microorganisms struggle to colonize the avian gut. As a result, the efficacy of non-native, environment-derived fiber-degrading microorganisms remains limited in poultry. Priorities include screening for highly efficient autochthonous strains from the avian intestinal tract (e.g., the cecum) and systematically elucidating the dynamic mechanisms underlying the degradation of diverse fiber substrates within the digestive tract. At the same time, priority should be given to conducting dose-titration studies, routinely reporting fermentation parameters and stability data and validating the reproducibility of results through multi-center trials.

Overall, most poultry studies used corn-soybean meal basal diets and employed solid-state fermentation or direct probiotic supplementation, with endpoint indicators covering digestive enzyme activity, intestinal morphology, microbiota composition, antioxidant capacity, egg quality and growth performance. Although multi-dosage designs were adopted, common limitations across most studies included opaque fermentation parameters, lack of batch-to-batch stability and failure to analyze fermentation products. Due to the short digestive tract and rapid digesta transit in poultry, exogenous strains are difficult to colonize. Future work should prioritize the isolation of host-derived autochthonous strains and systematic documentation of fermentation conditions, as well as cross-site trials to rigorously verify experimental reproducibility.

### 4.3. The Application of Fiber-Degrading Microorganisms in Ruminant Production

Fiber-degrading microorganisms play a central role in the efficient utilization of fiber by ruminants. Current research has largely focused on screening highly efficient strains from native niches such as the rumen and exploring their probiotic potential [64]. Despite considerable variations in strain sources, dosages and outcome measures across studies, the findings consistently converge on a “microbiota–immunity–barrier” multi-target regulatory mechanism. Through this mechanism, specific probiotics or their metabolites systematically enhance growth performance and gut health in ruminants. Unlike aerobic fungi, anaerobic fungi are native rumen inhabitants that physically penetrate plant cell walls via zoospores and rhizoids and contain hydrogenosomes (producing H_2_, interacting with methanogens), forming a unique dual degradation mechanism (physical penetration and enzymatic hydrolysis). They assemble cellulosome complexes via cohesin–dockerin interactions for efficient cellulose degradation. For growth promotion, probiotics primarily exert their effects by optimizing ruminal fermentation and nutrient utilization. Previously, a study demonstrated that dietary supplementation with *Aspergillus oryzae* culture (especially at 2 g/d) significantly increases the abundances of total anaerobic bacteria, fiber-degrading bacteria and fungi in the rumen and elevates concentrations of total short-chain fatty acids and major volatile fatty acids while concurrently decreasing ammonia nitrogen levels. This effectively enhances dry matter intake and apparent nutrient digestibility, thereby improving ruminal fermentation function [65]. Although the multi-dosage design (1, 2 and 3 g/d of *Aspergillus oryzae* culture) in this study enhanced reproducibility and dose–response reliability, it did not assess the production performance parameters such as milk yield and composition. Antanaitis et al. [66] demonstrated that daily supplementation with *Bacillus subtilis* at 7.5 mL for 90 days pre-weaning significantly improved the metabolic status of calves. Specifically, it reduced aspartate aminotransferase activity by 41.12%, while increasing γ-glutamyl transpeptidase activity and serum phosphorus concentration by 64.68% and 9.36%, respectively. This intervention effectively promoted growth and development, with body weight increased by 4.11%, 3.75% and 2.91% at 30, 60 and 90 days of age, respectively, thereby enhancing growth performance and metabolic health in neonatal calves. The two studies described above used a fungal culture and a bacterial probiotic, respectively, with markedly different dosages and treatment durations, yet both achieved growth improvement, suggesting that different microbial preparations may produce similar productive benefits through distinct mechanisms. Furthermore, Zhang et al. [67] evaluated the potential of *Bacillus amyloliquefaciens* fsznc-06 and *Bacillus subtilis* fsznc-09 to effectively promote digestive system development and modulate gut microbiota in weaned black goats. Following supplementation, ruminal papillae and small intestinal villus morphology were significantly improved in the lambs. The richness and diversity of the ruminal and cecal microbiota were enhanced, the abundance of beneficial bacteria was increased and the relative proportion of pathogenic bacteria was decreased. Functional analysis further demonstrated that *Bacillus* intervention strengthened the contributions of the microbial community to host metabolism and physiological regulation. Compared with the above studies focusing on ruminal fermentation and metabolic status, this study provides more detailed intestinal morphology and microbiota data. Although its reproducibility is high (clearly defined strains, multi-parameter validation), the experimental period was short (only 30 days), and production performance (daily gain and feed intake) was not assessed.

In terms of improving intestinal health, fiber-degrading microorganisms act through far more diverse and systematic mechanisms. Cidan et al. [68] characterized *Enterococcus faecalis* JM263, isolated from yak intestines, which exhibits high-efficiency cellulose-degrading activity and excellent probiotic properties. This strain effectively degrades straw, displays strong gastrointestinal tolerance (75.3% survival rate in gastric juice), and shows remarkable antibacterial and antioxidant capacities (inhibition zone = 21.27 mm, free radical scavenging rate >36%). It significantly optimizes ruminal fermentation by increasing SCFA production, lowering ammonia nitrogen concentrations, and promoting the proliferation of beneficial bacteria, highlighting its potential as a ruminant-specific probiotic. However, based only on correlational analysis and low-resolution 16S rRNA sequencing, this study failed to establish causality between JM263-induced SCFA alterations and microbiota remodeling or to differentiate core functional bacteria from passive passengers. Thus, the mechanism of action of JM263 remains to be validated. Furthermore, a previous experiment confirmed that dietary supplementation with *Bacillus coagulans* 315 (5 × 10^8^ CFU/d) effectively alleviates diarrhea in lambs. It acts by enriching beneficial bacteria (*Enterococcus* and *Lactobacillus*), suppressing the growth of pathogenic taxa (*Campylobacter* and *Escherichia coli*), and optimizing gut microbiota composition. Meanwhile, it downregulates the expression of pro-inflammatory cytokines (TNF-α, IL-1β and IL-6), upregulates anti-inflammatory cytokine expression and elevates intestinal immunoglobulin levels and antioxidant enzyme activities, thereby enhancing intestinal immune function and strengthening the mucosal barrier [69]. Although the multi-dosage design improved reproducibility and dose–response reliability, this study was limited to a short-term trial (14 d) and long-term effects are unknown. Compared with the studies above, a duration of 14 days represents an important limitation for assessing practical application.

Overall, ruminants generally show more robust responses to fiber-degrading microorganisms than monogastrics, likely due to longer gastrointestinal retention time and a favorable rumen environment. However, most studies were conducted in calves or lambs, and their generalizability to mature animals remains unclear. Dosages vary by more than 1000-fold across studies, yet none has established a dose–response curve. Although anaerobic fungi hold great potential in ruminant nutrition, they have long been neglected due to difficulties in strict anaerobic cultivation and the lack of genetic manipulation systems. Developing feasible cultivation methods and validating their efficacy through in vivo trials should be prioritized. More fundamentally, in vitro degradation assays cannot simulate the dynamic gastrointestinal environment, host immunity or microbial interactions, nor can they adequately assess colonization capacity, dose–response relationships, or long-term safety. Therefore, in vitro results should be interpreted only as preliminary screening findings and must be ultimately validated through in vivo animal experiments.

## 5. Existing Problems and Prospects

**Figure 2:**
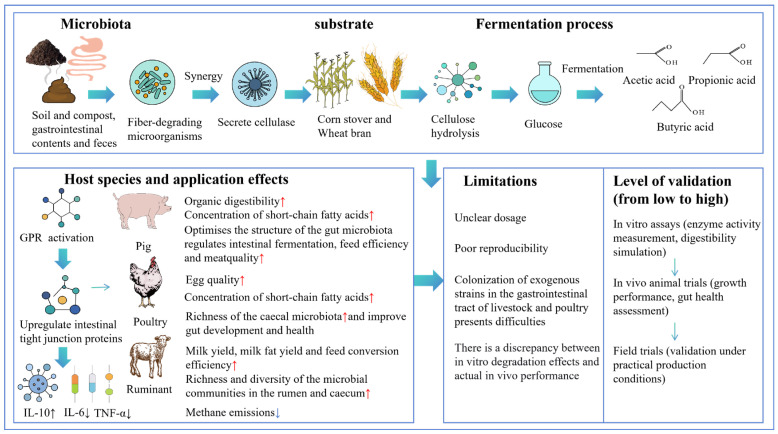
The application of fiber-degrading microorganisms in livestock and poultry.

As shown in Figure 2, fiber-degrading microorganisms have been demonstrated under laboratory conditions to convert cellulose in agricultural waste into sugars that can be utilized by livestock and poultry, which is of great significance for improving feed efficiency and enhancing gut health in controlled animal trials. However, evidence for reducing reliance on traditional feed under practical production conditions remains limited. Furthermore, the transition from laboratory research to industrial application still faces multiple bottlenecks. Critically, most current positive findings are derived from correlational observations, short-term experiments, or in vitro assays and await in vivo validation, a limitation that runs through many of the studies cited in this review. Urgent breakthroughs are required in the following areas.

(1) Poor adaptability of microbial strains to the gastrointestinal environment. Highly efficient strains are mostly isolated from soil and compost (e.g., *Trichoderma* and *Aspergillus*), making it difficult for them to survive and colonize the acidic, anaerobic and rapid-transit intestinal environment of livestock and poultry. Currently, the industry standard relies on spore-forming bacteria (e.g., *Bacillus subtilis*) for their inherent tolerance, along with random mutagenesis for strain improvement. Both are well-established practices. To address this issue, future efforts should focus on mining indigenous strains from targeted digestive habitats such as the cecum and rumen, elucidating the molecular mechanisms underlying their colonization using multi-omics technologies, and employing synthetic biology tools to perform targeted engineering of colonization-related genes (e.g., acid tolerance, adhesion proteins). For example, CRISPR-Cas9 has been used to knock out sporulation genes in *Bacillus subtilis* to redirect metabolic flux toward enzyme production, and promoter engineering can enhance acid tolerance. These approaches offer more precise outcomes than traditional random mutagenesis, rather than simply pursuing the construction of “super engineered strains”. It should be noted, however, that these advanced strategies have currently only been validated in model systems; their efficacy in the actual livestock and poultry gut environment has not yet been demonstrated, and they remain distant from practical industrial application for feed strains, representing more of a medium- to long-term research goal.

(2) The single-strain approach neglects synergistic degradation driven by microbial consortia. Conventional screening efforts focus on individual strains, yet cellulose degradation is inherently a collaborative, multispecies process. As a result, using single strains to treat complex lignocellulosic wastes often leads to unstable and inconsistent outcomes. Against the backdrop of the current industrial landscape, single-strain products remain the mainstream in the market due to constraints from regulatory barriers and production controllability limitations, and this status quo is unlikely to shift in the near term. In response, modular synthetic consortia of fungi and bacteria should be constructed based on a metagenomics-informed, function-guided design principle: first, characterize the structural features of cellulose in the target waste material; then, combine strains with complementary enzyme production capabilities (e.g., *Trichoderma* providing exoglucanases and *Bacillus* providing endoglucanases). Concurrently, efforts to mine uncultured microbial resources should be strengthened by leveraging metagenome-assembled genomes and single-cell sequencing technologies to obtain genomic information from fiber-degrading microorganisms that have not yet been cultured, thereby expanding the strain resource repository. It should be noted, however, that these strategies, while conceptually attractive, are still in early-stage exploration and have not yet resulted in standardized workflows or commercial precedents.

(3) Spatio-temporal mismatch between the release of degradation metabolites and host metabolic processes. Marked variations exist in sugar absorption capacity among different livestock and poultry species. The rapid release of reducing sugars can be readily competed for by non-target microbial communities, leading to abdominal bloat or diarrhea. In current practice, basic formulation adjustments and the use of simple protective carriers are feasible measures and have been applied to some extent. As a first step toward precision formulation, a preliminary substrate–microbiota–host knowledge base should be established for different livestock and poultry species to systematically determine sugar release kinetics of various microbial strains on different fiber substrates and match these data with host intestinal absorption capacities. This knowledge base could evolve into a predictive database in the long term, but for now it remains a long-term vision and is not yet an available tool. With sufficient accumulation of standardized data, this knowledge base is expected to eventually develop into a predictive compatibility database. In terms of formulation design, the concept of drug delivery technology could inspire the future development of controlled-release inoculants, but such approaches remain at an early research stage due to technical complexity and high production costs. In the short to medium term, more practical improvements, such as optimizing carrier materials or applying microencapsulation for basic protection, are more feasible to enhance strain survival in the gut. Unified in vitro–in vivo evaluation models should also be established to predict the actual efficacy of different formulations. This need is clear, but no existing solution is available, and the development of such models itself represents an urgent research task.

(4) Lack of safety assessment and industrialization standards. Against the backdrop of growing global concerns over antimicrobial resistance (AMR) and feed biotechnology safety, systematic evaluations are lacking for risks associated with fungal sensitization, mycotoxin production, and resistance gene transfer. Heavy metals and mycotoxins may accumulate in waste substrates, enzyme activity assays lack unified methodologies, and microbial inoculants exhibit poor stability. However, existing regulatory frameworks, including the European Food Safety Authority Panel on Additives and Products or Substances used in Animal Feed guidance, the United States Generally Recognized as Safe system, and the European Food Safety Authority Qualified Presumption of Safety approach, already provide well-established pathways for assessing the safety of feed microorganisms. At present, no universally accepted safety assessment framework or industrialization standard specifically targets fiber-degrading microorganisms for animal feed. For safety assessment, a three-tier evaluation system should be established: whole-genome safety screening (virulence factors, resistance genes, toxin genes), in vitro cytotoxicity testing and in vivo animal safety evaluation. These tiers align with requirements already specified in existing frameworks: the European Food Safety Authority requires whole-genome sequencing for virulence and resistance genes, minimum inhibitory concentration determination for antibiotics, and in vivo toxicity studies; the United States Generally Recognized as Safe system demands strain-specific evidence of safety, including absence of pathogenicity, antibiotic resistance, and toxigenic potential; and the European Food Safety Authority Qualified Presumption of Safety grants presumptive safety status at the taxonomic level based on four criteria, namely, taxonomic identity, absence of pathogenicity, absence of transmissible antibiotic resistance, and absence of toxigenic potential. Specific standards may refer to the Codex Alimentarius Commission requirements for strain identity confirmation; the guidelines of the European Food Safety Authority Panel on Additives and Products or Substances used in Animal Feed on minimum inhibitory concentration determination, toxigenic gene detection and pathogenicity testing; and the European Food Safety Authority Qualified Presumption of Safety assessment framework (taxonomic identity, absence of pathogenicity, absence of transmissible antibiotic resistance and absence of toxigenic potential). To address strain stability, industry standards should be developed to unify cellulase activity assays and to establish minimum viable counts, storage stability, and shelf-life standards for microbial inoculants, followed by process feasibility validation through pilot-scale fermentation and field trials. However, none of these frameworks have yet been fully implemented or reported with complete data specifically for fiber-degrading feed additives; rather than being conceptually lacking, they remain incompletely applied. Regarding the regulatory pathway, achieving reform goals such as dynamic updating of feed additive catalogs or establishing a biosafety negative list requires multi-stakeholder balancing and long-term coordination while also being constrained by the specific legal frameworks of different countries. In the short term, a more realistic priority is to harmonize safety assessment standards and define the tiered evidence packages that academia can submit to regulatory authorities to facilitate science-based evaluation decisions.

(5) Mechanistic understanding lags behind phenotypic observation. Although many studies report improved fiber digestibility or animal performance, the underlying mechanisms are often oversimplified, with effects commonly attributed solely to cellulase activity. It should be emphasized that improved fiber digestibility may involve multiple factors, including gut microbiota changes, feed matrix disruption, fermentation metabolites (e.g., SCFAs) and host physiological regulation. Moreover, deeper mechanisms such as LPMO contributions, substrate accessibility, cellulosome synergy and regulatory network dynamics remain largely unexplored. This represents the current state of the field, not a future prospect. Researchers should adopt a systems-level approach integrating metagenomics, metabolomics and host physiology, rather than focusing solely on single-enzyme assays. Systematic CAZyme profiling of candidate strains using standardized methods should also be prioritized, along with exploring the potential of LPMOs to enhance in situ fiber degradation. In practice, however, no study has yet performed such an integrated analysis, from strain characterization to in vivo mechanistic validation, specifically for fiber-degrading feed additives. Most multi-omics studies remain descriptive and correlative.

(6) Lack of direct comparisons between fungal enzymes and bacterial probiotics. As initially noted in Section 2.2, a critical gap in the current literature is the absence of direct and head-to-head comparisons between fungal enzyme preparations and bacterial probiotics under identical experimental conditions. While both approaches have been individually studied, few studies have systematically compared their efficacy using the same animal model, diet, dosage rationale and outcome measures. This gap represents a major obstacle for evidence-based feed formulation. Unlike many other proposals that remain conceptual, this is a clearly defined and immediately actionable research priority. Priorities for further work include such comparative trials to determine which approach or combination is more effective and cost-efficient for specific applications.

Thus, while fiber-degrading microorganisms have significant potential to improve animal feed utilization, current evidence is largely limited to correlational data, short-term experiments and in vitro assays. The poor reproducibility of translating in vitro findings to in vivo animal conditions represents a critical bottleneck in the field. As noted throughout this section, many of the proposed advanced approaches remain conceptual. To translate these findings from proof-of-concept to practical application, the field must pivot toward rigorous in vivo validation, standardized protocols and systematic reporting of negative results while keeping a clear distinction between near-term feasible improvements and long-term conceptual directions. Therefore, future research should prioritize improving the reproducibility of in vitro–in vivo translation through standardized experimental designs, multi-center validation trials and mandatory reporting of negative results.

## 6. Industrial-Scale Fermentation and Commercialization Challenges

Translating fiber-degrading microorganisms from laboratory findings into commercial feed additives faces major challenges in industrial fermentation, product formulation and market entry. At the industrial scale, filamentous fungi increase broth viscosity, causing oxygen transfer limitations and reduced enzyme yields; batch-to-batch variability in raw substrates undermines product consistency; downstream processing accounts for 30–50% of fungal enzyme costs; and feed pelleting (70–90 °C) inactivates 50–90% of fungal enzymes, while bacterial spores survive but show delayed germination. Regarding commercial products, fungal enzyme preparations suffer from poor thermostability and low gut survival, bacterial probiotics show strain-dependent effects without direct comparisons and no published study has directly compared fungal enzymes versus bacterial probiotics under identical conditions, leaving feed formulators without evidence-based guidance; regulatory approval also varies substantially across regions (China, EU, US), discouraging global commercialization. To overcome these barriers, research should develop standardized fermentation protocols, adopt accelerated stability testing, and conduct multi-site field trials; technological innovations such as encapsulation (e.g., spray-drying with protective matrices) and synthetic biology tools, including promoter engineering (to boost enzyme production), CRISPR interference (to repress negative regulators) and heterologous expression of heat-shock proteins, can enhance strain robustness, and regulatory pathways should establish tiered approval frameworks for Qualified Presumption of Safety-status genera, develop cost-effectiveness benchmarks, and create public databases of validated strains with standardized phenotypic data.

## Figures and Tables

**Table 1:** The main stages and key characteristics of cellulose degradation by *Bacillus* species.

Stages	Processes	Key Molecules/Structures	Key Features
①	Signal detection	Carbon source limitation and presence of cellulose	*Bacillus* perceives multiple environmental signals (e.g., carbon source limitation and cellulose availability) and integrates them via complex regulatory networks to initiate the degradation process
②	Intracellular regulation	NADH, calcium signaling pathways, transcriptional regulators (SigI, CcpA, LacI and Rok), cellulase genes (bglC, bglH and cel9A)	Hierarchical regulatory networks: carbon catabolite repression (CCR) via CcpA prioritizes easily metabolizable sugars; σ-factor switching (SigI and SigD) directs RNA polymerase to specific promoters; post-translational modulation (phosphorylation, allostery) fine-tunes enzyme activity; feedback loops and cross-regulation integrate carbon and energy status
③	Transcription and Translation	RNA polymerase, ribosomes, DNA → mRNA → enzyme proteins	Synthesis of a cellulase protein precursor containing a signal peptide
④	Protein secretion	Secretory system and signal peptide	Transporting cellulase across the membrane to the extracellular space
⑤	Cellulose degradation	Endoglucanase, cellobiohydrolas and β-glucosidase	Three types of enzymes work together to completely break down cellulose into glucose

**Table 2:** Comparative analysis of fungal and bacterial cellulase systems in animal feed applications.

Comparative Aspect	Fungi (*Trichoderma*, *Aspergillus*)	Bacteria (*Bacillus*, *Streptomyces*)
Enzyme system type	Free extracellular enzymes	Free enzymes or cellulosomes
Cellulosome presence	No	Yes (in some species)
Enzyme yield	>100 U/mL	<80 U/mL
pH optimum	Acidic (pH 4–5)	Wide (pH 5–9)
Temperature optimum	40–50 °C	30–60 °C (strain-dependent)
Industrial production maturity	Mature; large-scale fermentation established	Developing; spore-based production scalable
Production cost	Moderate	Varies by strain, process, and formulation: low for crude enzyme broth; high for purified enzymes or specialized strains
Storage stability	Liquid enzymes: moderate (limited stability under cold storage)	Varies by formulation: high for spore-based (long-term stable at room temperature); low for vegetative cells or liquid enzymes (require a cold chain)
Regulatory status	Generally recognized as safe (enzymes)	Strain-dependent (probiotics)
Gut survival	Poor (degraded in GI tract)	Good (spore-forming)
Probiotic potential	No	Yes
Primary safety concerns	Mycotoxin contamination; allergenicity	ARG transfer risk
Main application in feed	Feed pretreatment (off-feed)	Direct-fed microbials (in-feed)

**Table 3:** Comparison of screening methods and host-specific considerations for cellulase application.

Category	Subcategory	Key Features	Advantages	Limitations	Recommended Applications
Screening methods	Culture-based (Congo red)	Visual clear zones; culture-dependent	Simple; low cost; yields isolates	Low throughput; misses most diversity	Initial strain isolation
	Metagenomics and metatranscriptomics	Culture-independent; CAZyme annotation	High throughput; accesses unculturable microbes	Genetic potential does not equal actual activity	CAZyme discovery; functional prediction
	Metaproteomics	Direct activity screening; protein evidence	Functional validation; direct evidence	Technically complex; heterologous expression challenges	Enzyme discovery; mechanism validation
Host species	Pigs (hindgut fermenter)	Retention 24–48 h; no endogenous cellulase	Fermentation pretreatment effective	Gastric acid kills microbes; hindgut colonization difficult	Fermented feeds; encapsulated probiotics
	Poultry (short gut)	Retention 2–4 h; spore-formers preferred	Enzyme cocktails act quickly	Non-spore-formers fail to colonize	Spore-based probiotics; enzyme cocktails
	Ruminants (foregut fermenter)	Retention 48–72 h; natural rumen synergy	High compatibility with resident flora	Competition with native microbes; variable dosage	Live cultures; fungal fermented products

## Data Availability

No new data were created or analyzed in this study. Data sharing is not applicable to this article.

## Abbreviations

The following abbreviations are used in this manuscript:

AA	Auxiliary Activities
AMR	Antimicrobial Resistance
CMC	Carboxymethyl Cellulose
CFU	Colony-Forming Unit
CMC-Na	Sodium Carboxymethyl Cellulose
CON	Control
CE	Carbohydrate Esterases
DNA-SIP	DNA-Stable Isotope Probing
DDP	Degraded Date Pit powder
FF	Enterococcus faecium
FL	Fermented product of *Laetiporus sulphureus*
GH	Glycoside Hydrolases
HA/FA	fulvic acid
PRO	Probiotics
PL	Polysaccharide Lyases
LPMO	Lytic Polysaccharide Monooxygenase
MAG	metagenome-assembled genome
SCFA	short-chain fatty acids
